# Phagocytosis of Advanced Glycation End Products (AGEs) in Macrophages Induces Cell Apoptosis

**DOI:** 10.1155/2017/8419035

**Published:** 2017-12-20

**Authors:** Yuan Gao, Hidenori Wake, Yuta Morioka, Keyue Liu, Kiyoshi Teshigawara, Megumi Shibuya, Jingxiu Zhou, Shuji Mori, Hideo Takahashi, Masahiro Nishibori

**Affiliations:** ^1^Department of Pharmacology, Okayama University Graduate School of Medicine, Dentistry and Pharmaceutical Sciences, Okayama 700-8558, Japan; ^2^Department of Pharmacology, School of Pharmacy, Shujitsu University, Okayama 703-8516, Japan; ^3^Department of Pharmacology, Faculty of Medicine, Kinki University, Osakasayama 589-8511, Japan

## Abstract

Advanced glycation end products (AGEs) are the products of a series of nonenzymatic modifications of proteins by reducing sugars. AGEs play a pivotal role in development of diabetic complications and atherosclerosis. Accumulation of AGEs in a vessel wall may contribute to the development of vascular lesions. Although AGEs have a diverse range of bioactivities, the clearance process of AGEs from the extracellular space, including the incorporation of AGEs into specific cells, subcellular localization, and the fate of AGEs, remains unclear. In the present study, we examined the kinetics of the uptake of AGEs by mouse macrophage J774.1 cells *in vitro* and characterized the process. We demonstrated that AGEs bound to the surface of the cells and were also incorporated into the cytoplasm. The temperature- and time-dependent uptake of AGEs was saturable with AGE concentration and was inhibited by cytochalasin D but not chlorpromazine. We also observed the granule-like appearance of AGE immunoreactivity in subcellular localizations in macrophages. Higher concentrations of AGEs induced intracellular ROS and 4-HNE, which were associated with activation of the NF-*κ*B pathway and caspase-3. These results suggest that incorporation of AGEs occurred actively by endocytosis in macrophages, leading to apoptosis of these cells through NF-*κ*B activation.

## 1. Introduction

AGEs (advanced glycation end products) are the products of a series of nonenzymatic modifications of proteins by reducing sugars such as ribose, glucose, or glucose-6-phosphate that are related to processes involved in some progressive vascular aging diseases. Long-term incubation of proteins with glucose leads to advance glycation end products (AGEs) through the formation of early products such as Schiff bases and Amadori products. This process also exists in our body because every day we ingest such compounds in our diet [1]. In a healthy body, homeostasis controls these processes to maintain steady state or dynamic stability. However, in diabetic patients, high levels of blood glucose induce continuous irreversible glycation and oxidation of proteins and lipids, which lead to the formation of AGEs [2].

There is a plethora of evidence linking inflammation to the pathogenesis of vascular complications in diabetes. The formation of AGEs in arterial wall proteins such as the extracellular matrix may be responsible for accelerated atherosclerosis and microvascular disorders. One of the ways AGEs exert their cellular effects is by binding to receptors on the cell surface, such as the receptor for AGE (RAGE), lactoferrin-like polypeptide (LF-L), and scavenger receptor-A on macrophages [3–5]. Recent studies have revealed that the interaction of AGEs with RAGE receptors evokes oxidative and proapoptotic reactions in various types of cells and is thus involved in the pathogenesis of vascular complications in diabetes [6]. Apart from the receptor pathway, the accumulation of AGEs may be accompanied by protein cross-linking and cell damage. It has been reported that administration of an AGE aptamer significantly suppressed inflammatory cytokine expression in the kidneys of DM mice and prevented renal dysfunction by decreasing plasma levels of urea nitrogen and creatinine [7]. Although it is well known that AGEs are present in atherosclerotic plaques, especially in patients with diabetes, and that AGEs appear to be involved in macrophage foam cell formation, the precise mechanisms of their roles in these contexts are poorly understood [8]. Thus, it is important to understand the fate of endogenously produced AGE proteins and how they are eliminated from circulation in the homeostatic condition. There is evidence that macrophages play a pivotal role in eliminating AGEs and maintaining homeostasis by scavenger-receptor-mediated endocytosis in hepatic sinusoidal Kupffer and endothelial cells [9]. However, we still do not know the exact mechanism by which macrophages clear AGEs and the cell fate of macrophages after exposure to AGEs.

In the present study, our observations focused on the incorporation of AGEs in macrophages and the subcellular localization of AGEs. The results revealed that the incorporation of AGEs and the subsequent fate of AGEs in macrophages are dynamic processes and that caspase-3-dependent cell death is essential to the pathophysiological nature of these processes.

## 2. Materials and Methods

### 2.1. Reagents

AGEs were produced at our laboratory using BSA from Sigma (St. Louis, MO) as substrate proteins. The antibodies against AGEs were raised according the method of Morioka et al. [10] as mentioned below. The following reagents were purchased from commercial sources: RPMI-1640 medium (Sigma); penicillin-streptomycin solution (Sigma); 200 mM L-glutamine (Invitrogen, Carlsbad, CA); fetal bovine serum 16000 (Invitrogen); PBS, TBS and formaldehyde solution (Sigma); 10 mM Tris-buffered saline and formaldehyde solution (Katayama Chemical Industries, Osaka, Japan); and Triton X-100 (Nacalai Tesque, Kyoto, Japan). Normal goat serum, normal rabbit IgG, AlexaFluor 488-labeled goat anti-rabbit IgG (H+L), and AlexaFluor 555 goat anti-mouse IgG (H+L) were obtained from Abcam (Cambridge, UK); rabbit-caspase-3 antibody from Cell Signaling (Danvers, MA); anti-NF-*κ*B p65 antibody (Rb pAb; 16502) from Abcam; antiphosphatidylserine antibody from Upstate Biotechnology (Lake Placid, NY); goat anti-rabbit IgG (HRP) and DAPI from Life Technology (Carlsbad, CA); and West Dura extended duration substrate, 2′,7′-dichlorodihydrofluorescein diacetate (H_2_DCF-DA), and nuclear and cytoplasmic extraction (NE-PER) reagents from Thermo Scientific (Waltham, MA).

### 2.2. Synthesis of AGEs

BSA was incubated under sterile conditions with 0.2 M glyceraldehyde (Sigma-Aldrich) and glycol aldehyde (Sigma-Aldrich), respectively, in 0.2 M phosphate buffer for 7 days (pH 7.4 at 37°C). AGE-2 and AGE-3 correspond to Glycer-AGEs and Glycol-AGEs, respectively. Each of the AGEs-BSA was dialyzed at 4°C to remove free aldehyde. The degree of glycation on BSA (AGE-BSA fluorescence) was measured by spectrofluorometric detection at excitation of 370 nm and emission of 440 nm.

### 2.3. Anti-AGE-Specific Polyclonal Antibody

Antibodies were produced from Japanese white rabbits. Rabbits were immunized with 5 ml AGEs-BSA emulsified with the same volume of Freund's complete adjuvant (Wako). The rabbits received first booster injection after three weeks, followed by two additional booster injections every week with emulsified Freund's incomplete adjuvant. After the whole blood was collected from the immunized rabbits under anesthesia, the antisera titers were determined by immunoblot analysis. Immunoglobulin fractions was purified using MEP HyperCel (Pall, Port Washington, NY, USA). Anti-AGE-specific antibodies were purified by using each of AGEs-BSA immobilized on Sepharose beads (GE Healthcare, Buckinghamshire, UK).

### 2.4. Cell Preparation

The murine cell line J774.1 (BALB/c) was purchased from JCRB Cell Bank (Osaka, Japan). Cells were incubated at 37°C in RPMI1640 medium (containing 10% FBS, 200 mM glutamine and 1% penicillin-streptomycin solution) in a 5% CO_2_ humidified atmosphere and passaged every 3-4 days. After three passages, the cell suspension (10^6^ cells/ml) was divided into 0.1 ml aliquots that were incubated for 2 hrs in wells of a 96-well plate; then the medium with FBS was removed. Cells with a new medium without FBS were coincubated in the presence or absence of AGEs (Falcon 353219 microtiter plate).

### 2.5. Immunofluorescence Detection of AGEs after Incorporation into J774.1 Cells

After incubation in the presence or absence of AGEs for 2 hrs, cells (10^5^ cells/well) were fixed with 10% formaldehyde for 20 min and then washed three times with PBS for 5 min each time. When the cell membranes were disrupted, 10 min incubation with 0.2% Triton X-100/TBS was performed; then, 3% normal goat serum (0.1% T-TBS) was used as a blocking agent for immunofluorescence detection. After 30 min, the samples were incubated with rabbit polyclonal anti-AGE-2 or anti-AGE-3, or normal rabbit IgG (1 : 250) at room temperature for 1 hr. After three washes in T-TBS, cells were incubated for 1 hr with a secondary antibody (AlexaFlour 488-labeled goat anti-rabbit IgG) at room temperature. The fluorescence intensity was determined by FlexStation 3 mutimode microplate reader within one hour after washing cells with 0.1% T-TBS three times. Before observing the cells, we changed the plate medium (0.1% T-TBS) into TBS (non-Triton X) solution. The cells were observed under fluorescent microscopy (Biozero BZ8000; Keyence, Osaka, Japan). The 3D pictures were taken by confocal microscopy (LSM 780 Zesis Jena, Germany).

### 2.6. Intracellular ROS Assay

Intracellular ROS activity was detected by 2′,7′-dichlorodihydrofluorescein diacetate (H2DCF-DA). An H2DCF-DA fluorescent probe is commonly employed and reacts with several ROS, including hydrogen peroxide, hydroxyl radicals, and peroxynitrite. We detected intracellular ROS after exposure of J774.1 cells to AGEs. The cells were plated at a concentration of 10^6^ cells/ml (1 × 10^5^ cells per well) into a 96-well plate. After stimulation by AGEs for 24 hrs, the cells were washed by PBS three times and loaded with 5 *μ*M H2DCFDA at room temperature in the dark for 15 min. The fluorescence was measured with excitation at 485 nm and emission at 510 nm. Fluorescence images of cells were taken by a BZ-X700 (Keyence, Osaka, Japan) fluorescence microscope.

### 2.7. Western Blot Analysis of Proteins

To analyze the cellular proteins, 6-well plates were used, with each well containing 1 ml of cell suspension (5 × 10^6^ cells/ml). AGE-2 and AGE-3 were diluted with PBS to give the final concentration of 20 *μ*g/ml or 100 *μ*g/ml. Cells were preincubated at 37°C for 1 hr, then stimulated with AGEs for 0, 1, 2, 4, 6, 8, 12, and 24 hrs. After obtaining cell pellets by centrifugation, cells were treated with SDS-PAGE sample buffer (10% SDS, 1% *β*-mercaptoethanol, 2% Tris-HCl (pH 6.8), 20% glycerin, 0.01% bromophenol blue, H_2_O). Proteins were separated by SDS-PAGE and electrophoretically blotted onto nitrocellulose membranes (Bio-Rad, Hercules, CA). After blocking with 1% BSA in PBS, the membranes were incubated with rabbit primary antibodies at 4°C overnight. After the incubation with goat anti-rabbit IgG (HRP), the protein bands were visualized by the luminal-based enhanced chemiluminescence (ECL) HRP substrate method (Thermo Fisher Scientific Inc.). An Image Quant LAS4000 system was used for detection.

### 2.8. Phosphatidylserine Staining

After stimulation with 100 *μ*l AGE-2 or AGE-3 for 12 hrs, J774.1 cells were fixed with 10% formaldehyde for 20 min and then washed three times for 5 min each in PBS. The buffer 0.2% Triton X-100/TBS could not be used because of the disruption of cell membrane. Blocking with 3% normal goat serum (0.1% T-TBS) for 30 min was performed before immunostaining. In these experiments, double immunostaining of cells with mouse anti-phosphatidylserine was performed. Alexa Fluor 555 goat anti-mouse IgG (H+L) and Alexa Fluor 488-labeled goat anti-rabbit IgG were used as secondary antibodies for double staining.

### 2.9. Statistical Analysis

Statistical significance was evaluated by ANOVA followed by Dunnett's test for multiple comparisons, or by Student's *t-*test for comparisons between two groups. *P* values less than 0.05 were considered significant.

## 3. Results

### 3.1. Uptake of AGEs in J774.1 Macrophages

**Figure 1:**
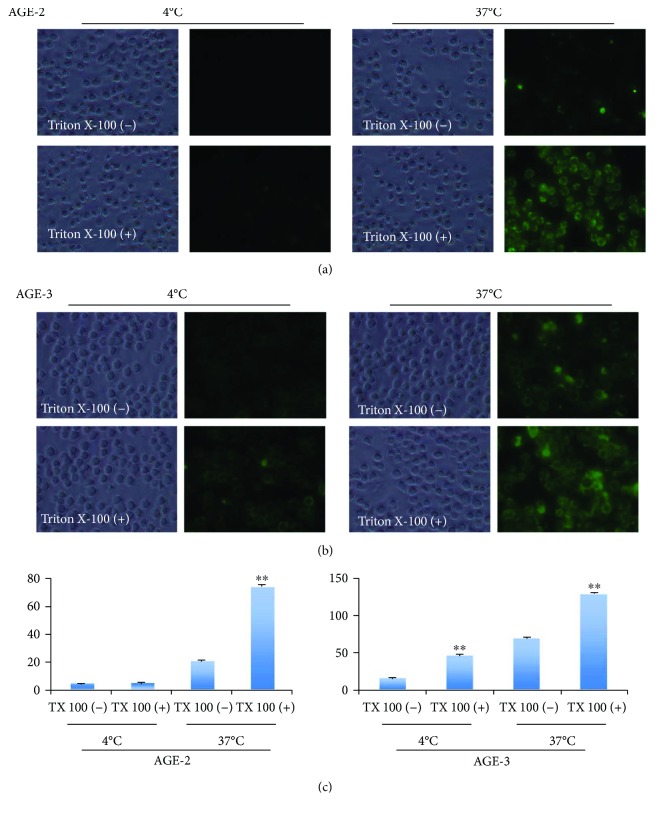
Uptake of AGE-2 and AGE-3 in macrophages. J774.1 cells (10^6^/ml, 100 *μ*l) were plated in 96-well black/clear plates in the culture medium without FBS. After adhesion was allowed for 1 hr, cells were incubated with BSA-AGE-2 or BSA-AGE-3 (20 *μ*g/ml) for 2 hrs. The incorporated BSA-AGE-2 or BSA-AGE-3 was detected by a specific polyclonal antibody made in our lab, and then a secondary antibody (AlexaFluor 488-labeled goat anti-rabbit IgG (H+L)) was added to obtain fluorescence. After fixation, the cells were treated with TBS in the presence or absence of 0.3% Triton X-100. Representative images are shown from four separate experiments. The fluorescence intensity was quantitated and the results are summarized in Figure 1(c). The results shown are means ± SEMs of four separate experiments. ^∗∗^ *p* < 0.01 versus Triton X-100 (−).

Figure 1 shows the temperature-dependent uptake of AGE-2 and AGE-3 into J774.1 macrophages at 2 hrs after the start of incubation. At 4°C, low levels of AGE binding were observed on the cell surface, irrespective of the presence or absence of Triton X-100 during the detection of antigens by the primary antibody. At 37°C, cell membrane-associated staining was apparent in the absence of Triton X-100, especially in the case of AGE-3, whereas the immunoreactivities for both AGE-2 and AGE-3 were enhanced in the cell-membrane-associated area and the intracellular compartment in the presence of Triton X-100. Thus, the fluorescence intensities of AGEs in the presence of Triton X-100 were significantly higher than those in the absence of Triton X-100 at 37°C. The quantitative determinations are summarized in Figure 1(c).

### 3.2. Time-Course and Concentration Dependency of the Intracellular Uptake of AGE-2 and AGE-3 and Their Subcellular Localization in Macrophages

**Figure 2:**
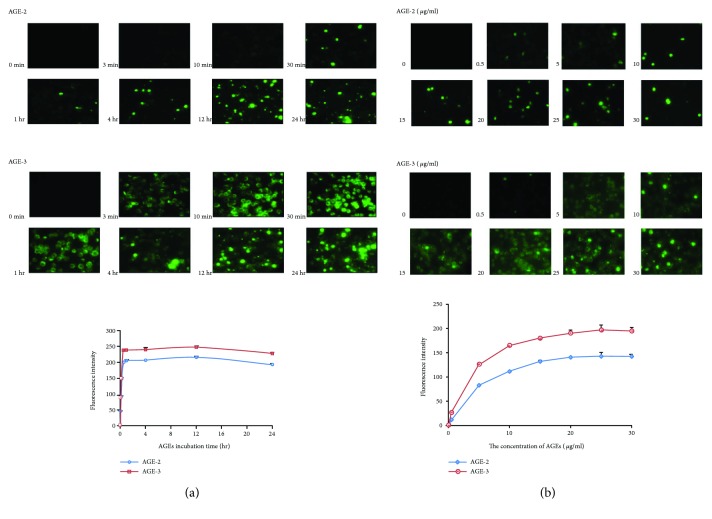
Time course and concentration dependency of uptake of AGE-2 and AGE-3 and their subcellular localization in macrophages. (a) The J774.1 macrophages were incubated with AGE-2 or AGE-3 (20 *μ*g/ml) at 37°C for the indicated periods. The AGE-2 or AGE-3 in the cells was detected by specific polyclonal antibodies as in Figure 1. Representative images from three separate experiments are shown. The fluorescence intensities were quantified with the results shown representing means ± SEMs of three separate experiments. (b) The J774.1 macrophages were incubated with different concentrations of AGE-2 or AGE-3 at 37°C for 1 hr. Representative images from three separate experiments are shown. The fluorescent intensities were quantified and results are shown as means ± SEMs of three separate experiments.

We determined time-course changes in the uptake of AGE-2 and AGE-3 in macrophages. The uptake of AGE-2 and AGE-3 was time-dependent and the fluorescence intensities of AGE-2 and AGE-3 increased up to 30 min. Although the fluorescence intensity of the cells was relatively constant thereafter, the distribution pattern of immunoreactivities was changed time-dependently and significantly with highly positive spots for both AGE-2 and AGE-3 (Figure 2(a)).  Figure 2(b) shows the concentration dependency of the uptake of AGE-2 and AGE-3 in macrophages at 30 min. The concentration-dependency curves for AGE-2 and AGE-3 showed a saturable shape, suggesting the existence of upper limits of capacity of incorporation.

**Figure 3:**
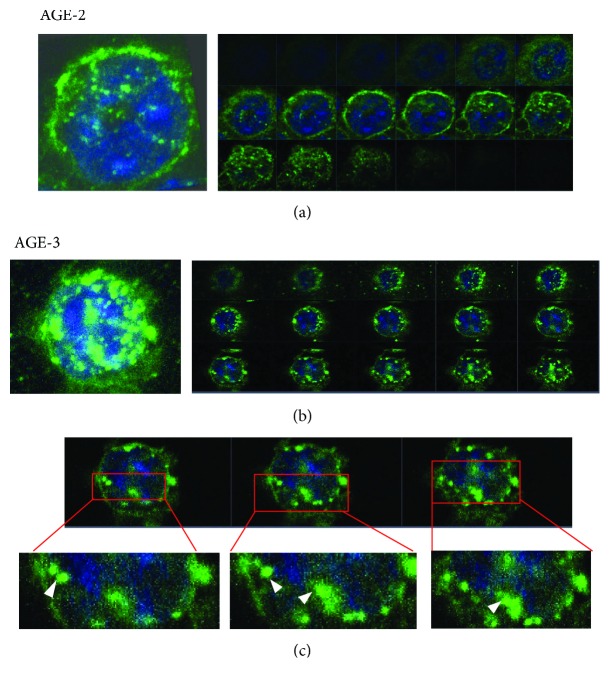
Three-dimensional images of J774.1 macrophages after AGE uptake. The J774.1 macrophages were incubated with 20 *μ*g/ml of AGE-2 (a) or AGE-3 (b) at 37°C for 2 hrs. The immunoreactivities in the cells (green) were observed under confocal microscopy with DAPI staining (blue). Serial sections in the *z*-axis are shown in the right-hand panels. Arrowheads in (c) indicate AGE-3-positive signals in the nuclear compartment.

Figure 3 shows typical pictures of AGE-2 and AGE-3 immunoreactivities at 2 hrs after the start of incubation with AGEs. The incorporated immunoreactivities formed small granule-like structures inside the cells, and some of the granules appeared to be present inside the nuclei (Figure 3(c)). The 3D pictures suggested that the interaction of AGEs with macrophages should include binding of AGEs to the cell surface, incorporation into the cytoplasm and nuclear compartments, and granule-like formation.  Figure 3(a) shows the distribution of AGE-2 in macrophages, with both cell-membrane binding and cytoplasmic incorporation.

### 3.3. Effects of Inhibitors of Endocytosis on Incorporation of AGEs in Macrophages

**Figure 4:**
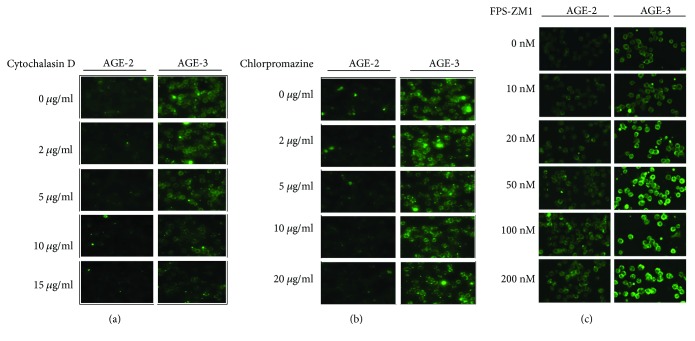
Effects of endocytosis inhibitors or RAGE inhibitor on AGE-2 and AGE-3 uptake in J774.1 macrophages. The J774.1 macrophages were preincubated with different concentrations of cytochalasin D (a), chlorpromazine (b), and FPS-ZM1 (RAGE inhibitor) (c) at 37°C for 1 hr. Then, the cells were incubated with AGE-2 or AGE-3 (20 *μ*g/ml) at 37°C for 30 min. AGE-2 or AGE-3 was detected as shown in Figure 1. Representative images from three separate experiments are shown.

To examine the mechanism of AGE uptake into macrophages, cells were preincubated with cytochalasin D, chlorpromazine, or FPS-ZM1 for 1 hr before the start of AGE uptake. The presence of cytochalasin D, an inhibitor of actin polymerization, blocked AGE-2 and AGE-3 uptake in macrophages (Figure 4). The inhibition by cytochalasin D was concentration-dependent and the inhibitory effect was significant at a lower concentration of 5 *μ*g/ml. However, chlorpromazine, an inhibitor of clathrin-dependent endocytosis, did not produce significant effects on incorporation of AGEs up to the concentration of 20 *μ*g/ml. As shown in Figure 4(c), the addition of FPS-ZM1, an antagonist of RAGE, enhanced the incorporation of both AGE-2 and AGE-3 in J774.1 cells in a concentration-dependent manner. The enhancing effects of FPS-ZM1 reached to maximal at 100 nM. The quantitative data on fluorescence intensities of incorporated AGEs were presented in Supplementary data 1.

### 3.4. Intracellular ROS Production during AGE Incorporation into Macrophages

**Figure 5:**
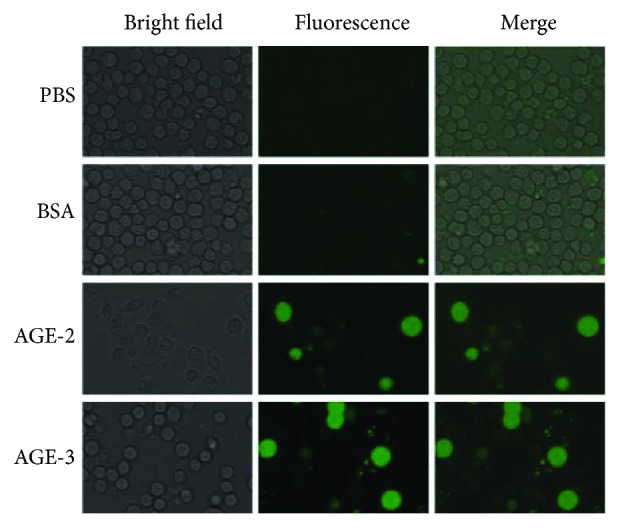
AGE-2 or AGE-3 stimulated ROS production in J774.1 macrophages. The J774.1 macrophages were incubated with 100 *μ*g/ml of AGE-2, AGE-3 or BSA at 37°C for 24 hrs. ROS production was detected by DCF fluorescence after the hydrolysis of DCF-AM inside the cells. Representative images from three separate experiments are shown.

ROS production in macrophages stimulated by AGEs was detected by DCF fluorescence after uptake of DCF-AM. Both AGE-2 and AGE-3 (100 *μ*g/ml) elicited ROS production strongly in some populations of cells, whereas the remainder of cells produced lower levels of ROS 24 hrs after the start of stimulation (Figure 5). In the PBS and BSA control groups, there were no detectable levels of ROS in the cells. As the bright-field image shows, the total numbers of cells were reduced in the groups exposed to AGE-2 and AGE-3.

### 3.5. Apoptosis of Macrophages after Uptake of AGE-2 and AGE-3

We next checked the apoptosis-inducing effects of AGEs because intracellular ROS production may be associated with the induction of apoptosis.

**Figure 6:**
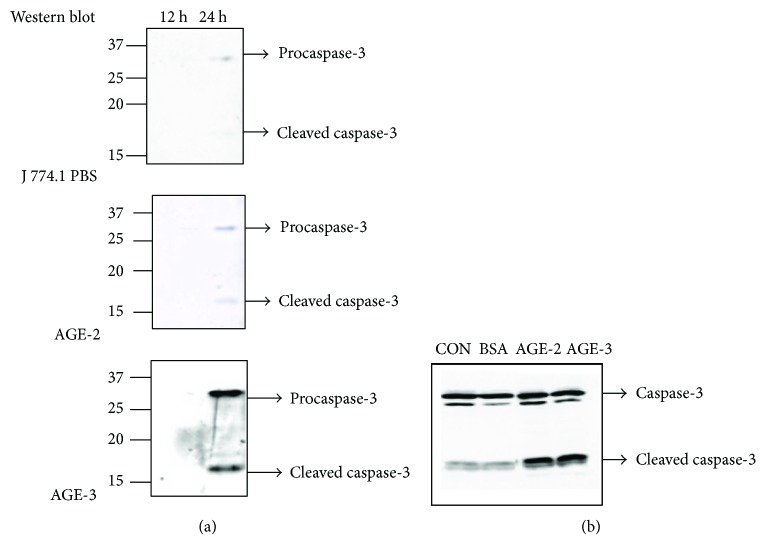
Western blot analysis of caspase-3 activation in J774.1 macrophages after stimulation with AGE-2 and AGE-3. The J774.1 macrophages were incubated with 20 *μ*g/ml (a) and 100 *μ*g/ml (b) of AGE-2, AGE-3, or BSA at 37°C for 12 and 24 hrs. Cleaved and uncleaved caspase-3 was detected by Western blotting.

Since the activation of caspase-3 is one of the markers of apoptosis, we determined the activated form of caspase-3 (cleaved caspase-3) on Western blotting (Figure 6). Prolonged incubation with AGE-2 and AGE-3 at 20 *μ*g/ml for 24 hrs significantly produced cleaved caspase-3 (Figure 6(a)). The cleaved caspase-3 in the AGE-2 group was faint compared with that in the AGE-3 group; however, the higher concentration of AGEs at 100 *μ*g/ml clearly showed that cleavage of caspase-3 was enhanced compared with the control groups (Figure 6(b)).

**Figure 7:**
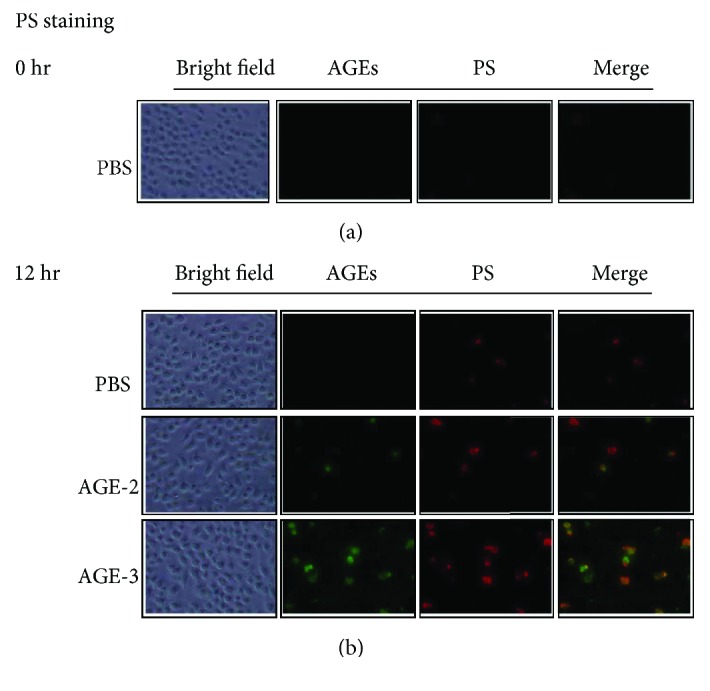
Expression of phosphatidylserine on the cell surface of J774.1 macrophages stimulated by AGE-2 and AGE-3. The J774.1 macrophages were incubated with 100 *μ*g/ml of AGE-2 or AGE-3 at 37°C for 12 hrs. Phosphatidylserine (PS) on the cellular surface was detected by anti-PS antibody. Merged pictures were obtained from AGE and PS staining. Arrowheads indicate double-staining cells.

As shown in Figure 7, incubation with AGE-2 and AGE-3 induced the expression of phosphatidylserine (PS) detected by annexin V binding on the cell surface 12 and 24 hrs after the start of incubation. The PS-positive cells were precisely merged with the cells exhibiting high AGE uptake (Figure 7).

These results showed that AGE-2 and AGE-3 induced cell apoptosis and that the ability of AGE-3 to induce cell apoptosis was stronger than that of AGE-2.

### 3.6. AGE-Induced 4-Hydroxynonenal (4-HNE) Production and Activation of the NF-*κ*B Pathway in Macrophages

**Figure 8:**
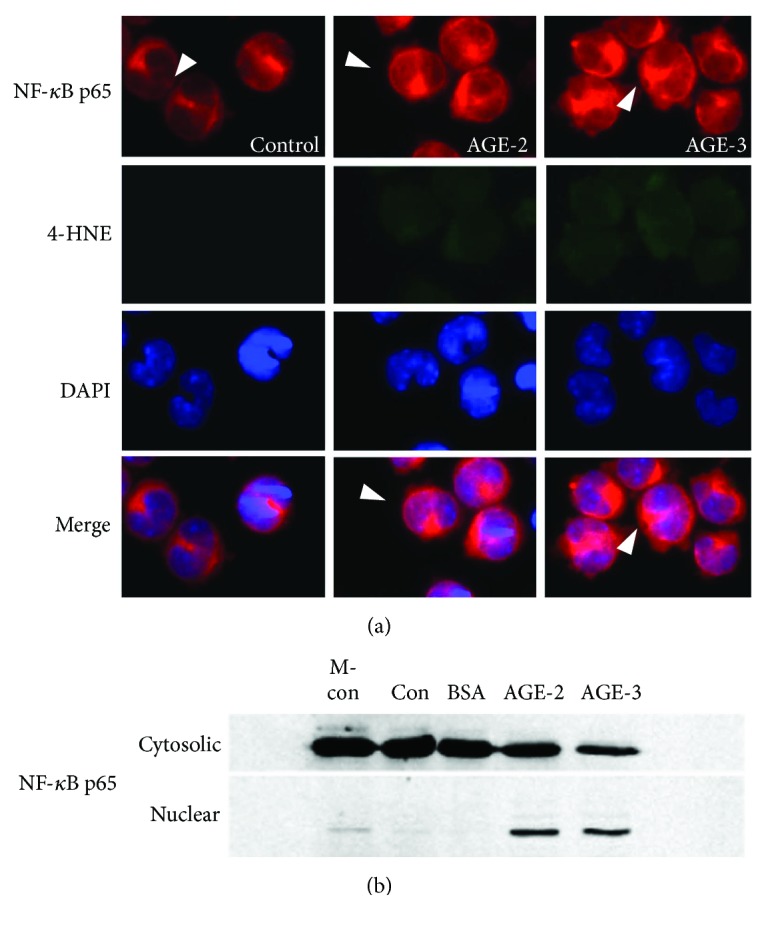
Double immunostaining of NF-*κ*B p65 and 4-hydroxynonenal after stimulation with AGE-2 and AGE-3 in J774.1 macrophages. The J774.1 macrophages were incubated with 100 *μ*g/ml of AGE-2 or AGE-3 at 37°C for 12 hrs. (a) The cells were stained with anti-NF-*κ*B p65 and anti-4-hydroxynonenal together with DAPI. White arrowheads show the relative expression of NF-*κ*B p65 in the nucleus after incubation with AGE-2 and AGE-3. (b) After incubation, the J774.1 macrophages were collected, and nuclear and cytosolic fractions were prepared for SDS-PAGE and NF-*κ*B p65 detection by Western blotting. Representative results from three independent experiments are shown.

Finally, we determined the translocation of NF-*κ*B p65 from the cytosolic to the nuclear compartment to evaluate NF-*κ*B activation.  Figure 8(a) shows that most of NF-*κ*B immunoreactivity in the control cells was located in the cytosolic compartment. However, the NF-*κ*B p65 immunoreactivity was partially translocated into the nuclear compartment after stimulation with AGE-2 and AGE-3. These immunocytochemical findings were consistent with the results of Western blotting, which showed the nuclear translocation of NF-*κ*B p65 by stimulation with AGE-2 and AGE-3 (Figure 8(b)). The cells stimulated with AGE-2 and AGE-3 also showed constant production of 4-HNE. This is consistent with ROS production during AGE uptake.

## 4. Discussion

AGEs have been suggested to be involved in the pathogenesis of many kinds of diseases, including diabetic complications, atherosclerosis, nephropathy, and neuropathic pain [11, 12]. The nonenzymatic production of AGEs occurs in the presence of higher concentrations of glucose in individuals with diabetes. Moreover, oxidative stress probably facilitates the formation of AGE adducts via the modification of proteins [13, 14]. There may be a diverse range of AGE subspecies produced, among which AGE-2 and AGE-3 exhibit strong toxic effects on specific cells [15, 16]. Receptors for advanced glycation end product (RAGE), SRA, LOX-1, and CD36 are the candidate receptors believed to mediate the various effects of AGEs [17]. Soluble RAGE treatment can cancel the effect of AGEs and be used as a biomarker in RAGE-dependent inflammation [18]. However, the mechanisms by which AGEs are cleared from the extracellular environment are inadequately understood. Therefore, we examined the fundamental uptake mechanism of AGEs into macrophages and its relevant effects on cell viability to better understand the fate of AGEs.

The present study clearly showed that J774.1 macrophages had the ability to incorporate AGE-2 and AGE-3 in a temperature-dependent and saturable manner. Then, we examined the effects of pretreatment with three inhibitors, cytochalasin D, chlorpromazine, and FPS-ZM1 [19, 20], on AGE-2 and AGE-3 incorporation in J774.1 cells. Cytochalasin D inhibits clathrin-independent endocytosis through depolymerizing F-actin. Chlorpromazine translocates chathrin and adaptor protein-2 from the cell surface to intracellular endosomes and inhibits clathrin-mediated endocytosis. The finding that cytochalasin D but not chlorpromazine inhibited AGE-2/3 uptake into J774.1 cells strongly suggests that AGE-2 and AGE-3 were taken up into macrophages mainly through clathrin-independent endocytotic mechanism but not through a clathrin-mediated mechanism. FPS-ZM1 is a high-affinity RAGE-specific blocker. In fact, we demonstrated that FPS-ZM1 blocked the binding of AGE-2 to sRAGE using surface plasmon resonance (Supplementary data 2). Surprisingly, FPS-ZM1 enhanced both AGE-2 and AGE-3 uptake into J774.1 cells. Since AGE-2 and AGE-3 has been demonstrated to be high-affinity agonists for RAGE [21, 22], the effects of FPS-ZM1 on AGE-2/3 uptake strongly suggest the inhibitory regulation by RAGE signaling on AGEs uptake mechanism. Thus, the signaling through RAGE may inhibit the clearance of AGE-2 and AGE-3, leading to the sustained presence of extracellular AGEs.

The expression of phosphatidylserine on the surfaces of macrophages increased in correlation with the amount of AGE-2 or AGE-3 the macrophages incorporated suggesting that apoptosis was induced after AGE incorporation. Caspase-3 is also activated in apoptotic cells both by extrinsic (death ligand) and intrinsic (mitochondrial) pathways; hence, the suggestion of apoptosis was confirmed by the detection of cleaved caspase-3 in cells after prolonged incubation with AGE-2 and AGE-3. It was shown in the present study that the uptake of AGEs was associated with ROS production and NF-*κ*B activation. Because we clearly detected the increased DCF fluorescence and NF-*κ*B translocation into nuclei at an early time point after AGE stimulation, these signaling pathways may be present upstream of caspase-3 activation, leading to cell apoptosis.

From the present study, it is postulated that macrophages are among the cells that remove AGEs from the extracellular environment. Previous studies suggested that AGEs might exist both extracellularly and intracellularly. Injection of aptamers produced against AGEs diminished the level of AGEs deposited in the glomerulus in diabetic rats, even though the exact mechanism was unclear [23]. In the present study, AGE-2 and AGE-3 are soluble ligands but are not bound to the extracellular matrix. Such soluble AGEs by themselves maybe readily taken up into macrophages from the extracellular environment.

Dense AGE immunoreactivities have been observed in atherosclerotic plaques in experimental animals as well as in human patients [24]. It is well known that these plaques contain numerous moribund foam cells. Thus, it is likely that AGEs bound to the cellular surface of foam cells induce oxidation stress, thereby promoting inflammatory cytokines and contributing to the expression of oxidized LDL (OxLDL) receptors in human monocyte-derived macrophages [25]. Our study implies that AGE incorporation and accumulation in macrophages in atherosclerotic plaques may be one of the factors leading to cell death. Oxidized-LDL has been known as an important factor that induces vascular endothelial cell dysfunction and phenotype changes in monocytes infiltrated into the intima. Since AGEs have been shown to induce gene expression of two important OxLDL receptors—macrophage scavenger receptor class A and CD36 [26, 27]—AGEs in atherosclerotic plaques could facilitate phenotype changes in macrophages in addition to the induction of apoptosis as observed in the present study. The main form of oxidized-LDL, which is taken up by LOX-1, was reported to be the aldehyde-modified form, suggesting a close similarity to AGEs [28, 29].

Another finding is the rapid production of 4-HNE after incorporation of AGEs. AGE-induced ROS production may induce 4-HNE productions in macrophages. Further works are necessary to identify the molecular signals involved in endocytosis of AGEs and the relationship between the signal-producing system and AGE incorporation in macrophages.

## 5. Conclusions

In conclusion, the present study clearly showed that macrophages incorporated toxic AGEs, AGE-2 and AGE-3, by a RAGE- or clathrin-independent endocytotic process. Uptake of AGE-2 or AGE-3 into cells may be associated with ROS production and NF-*κ*B activation. Accumulation of AGE-2 and AGE-3 inside macrophage cells in turn appears to trigger the activation of caspase-3, leading to apoptosis. These processes might in part reflect the events that occur in atherosclerotic plaques, especially with regard to foam cell death.

## Supplementary Materials

Supplementary 1 Effects of cytochalasin D and FPS-ZM1 on fluorescence intensities of AGE-2 and AGE-3 incorporated into J774.1 macrophages. Quantification of the fluorescence was done according to the description in Materials and Methods. ^∗^ *p* < 0.05 and ^∗∗^ *p* < 0.01 versus control.

Supplementary 2 Affinity of AGE-2 and RAGE in the presence or absence of FPS-ZM1, a RAGE antagonist. Biacore T200 technology was used to characterize the binding affinity of AGE-2 and RAGE. sRAGE (10 *μ*g/ml) was immobilized on the sensor chip (Biacore sensor chip CM5). (A) AGE-2 at a concentration of 0.125, 0.25, 0.5, 1, and 2 *μ*g/ml was used as flow protein over the surface. (B) Binding of AGE-2 (2 *μ*g/ml) to immobilized sRAGE in the absence or presence of FPS-ZM1 (100 nM). Blue line: AGE-2 alone; red line: AGE-2 + FPS-ZM1.
