# Supplementary material for: Phagocytosis of Advanced Glycation End Products (AGEs) in Macrophages Induces Cell Apoptosis
Source: Oxid Med Cell Longev. 2017 Dec 20;2017:8419035. doi: 10.1155/2017/8419035 (PMC5752849; doi:10.1155/2017/8419035)
Supplement: Supplementary 1 — Effects of cytochalasin D and FPS-ZM1 on fluorescence intensities of AGE-2 and AGE-3 incorporated into J774.1 macrophages. Quantification of the fluorescence was done according to the description in Materials and Methods. ∗ p < 0.05 and ∗∗ p < 0.01 versus control. [file 8419035.f1.docx]

Supplementary data 1
