# Supplementary material for: Phagocytosis of Advanced Glycation End Products (AGEs) in Macrophages Induces Cell Apoptosis
Source: Oxid Med Cell Longev. 2017 Dec 20;2017:8419035. doi: 10.1155/2017/8419035 (PMC5752849; doi:10.1155/2017/8419035)
Supplement: Supplementary 2 — Affinity of AGE-2 and RAGE in the presence or absence of FPS-ZM1, a RAGE antagonist. Biacore T200 technology was used to characterize the binding affinity of AGE-2 and RAGE. sRAGE (10 μg/ml) was immobilized on the sensor chip (Biacore sensor chip CM5). (A) AGE-2 at a concentration of 0.125, 0.25, 0.5, 1, and 2 μg/ml was used as flow protein over the surface. (B) Binding of AGE-2 (2 μg/ml) to immobilized sRAGE in the absence or presence of FPS-ZM1 (100 nM). Blue line: AGE-2 alone; red line: AGE-2 + FPS-ZM1. [file 8419035.f2.docx]

Supplementary data 2

A.

B.
